# The role of transplantation in Hodgkin lymphoma

**DOI:** 10.3389/fonc.2022.1054314

**Published:** 2023-01-26

**Authors:** Michael Maranzano, Monica Mead

**Affiliations:** Division of Hematology/Oncology, Department of Medicine, David Geffen School of Medicine, University of California Los Angeles, Los Angeles, CA, United States

**Keywords:** relapsed/refractory lymphoma, Hodgkin lymphoma (HL), autologous and allogeneic transplantation, autologous transplant (ASCT), allogenic transplant, targeted therapy, immunotherapy, maintenance therapy

## Abstract

Despite the success of frontline anthracycline-based chemotherapy for classical Hodgkin Lymphoma (cHL), approximately 15% of patients do not achieve an adequate response and require further therapy. For transplant-eligible patients, additional treatment followed by high-dose chemotherapy and autologous hematopoietic stem cell transplantation (autoHCT) provides a durable response in 50% of patients. The most refractory patients, including those requiring multiple lines of therapy to achieve a response or those relapsing after an autoHCT, may achieve long-term survival with allogeneic hematopoietic stem cell transplant (alloHCT). Contemporary salvage regimens used as a bridge to transplant have expanded to include not only non-cross resistant chemotherapy, but also brentuximab vedotin (BV) and checkpoint inhibitors (CPI). As the management of relapsed/refractory (R/R) cHL evolves with the introduction of novel agents, so too does the role of transplantation. The paradigm of chemosensitivity as a predictor for autoHCT efficacy is being challenged by favorable post- autoHCT outcomes in heavily pre-treated CPI-exposed patients. Contemporary supportive care measures, validated comorbidity assessments, and an increased donor pool with haploidentical donors have broadened the application of transplantation to an increasingly older and diverse patient population. Despite the introduction of increasingly effective treatment options for R/R cHL, transplantation continues to play an important role in the management of these patients. In this review, we explore the impact of salvage therapy on autoHCT, conditioning regimens, maintenance therapy and the diminishing role of alloHCT for patients with cHL.

## Introduction

Classical Hodgkin Lymphoma (cHL) is a B-cell malignancy featuring the diagnostic hallmark of multinucleated Reed–Sternberg cells found on pathologic exam of lymph nodes or other affected tissues. While 80% of patients with cHL achieve a durable remission with anthracycline-based combination chemotherapy in the frontline setting (1–4), 10-20% develop relapsed or refractory (R/R) disease after an initial complete response (CR) and 5-10% have primary refractory disease (5). Approximately 50% of fit patients with R/R cHL are cured with a platinum-based salvage regimen followed by high-dose chemotherapy and autologous hematopoietic stem cell transplantation (autoHCT) for responding disease (6, 7). In carefully selected patients who relapse after an autoHCT or with highly refractory disease, allogeneic hematopoietic stem cell transplantation (alloHCT) can provide a durable response leveraging a graft versus lymphoma (GVL) effect.

Substantial progress has been made in the treatment of cHL that has resulted in improved outcomes for patients with R/R disease. Phase 2 single-arm studies evaluating novel agents in R/R cHL including Brentuximab Vedotin (BV), a CD30-directed antibody- drug conjugate (FDA approval August 2011), and checkpoint inhibitors (CPIs) including nivolumab (FDA approval May 2016) and pembrolizumab (FDA approval March 2017), have shown CR rates as monotherapies of 32%, 16% and 28% respectively (8–10).

Despite broadening of the cHL therapeutic armamentarium, these novel agents as monotherapy are unlikely to result in cure; thus, hematopoietic stem cell transplantation continues to play an important role in the management of R/R disease. Use of combination chemotherapy, novel agents in earlier lines of therapy, immune-based therapy, and improvements in autoHCT, have resulted in a 10-year overall survival (OS) rate approaching 60% (11). With improved supportive care and incorporation of novel agents pre- and/or post-autoHCT, Center for International Blood and Marrow Transplant Research (CIBMTR) data reports substantial improvement in 3-year OS after autoHCT from 72% in the early 2000s to 92% for those recently transplanted 2016-2019 (12, 13).

## Patient selection for autologous transplant

Common frontline regimens for the treatment of cHL, including ABVD, BEACOPP, and more recently A-AVD, result in excellent outcomes (14, 15). Therefore, consolidative transplantation is rarely considered in first complete remission (CR1), supported by a randomized control trial (RCT). Carella et al. randomized 163 patients with unfavorable cHL in first response following 4 cycles of ABVD to an additional 4 cycles of ABVD or autoHCT (16, 17). There was no difference in 10-year overall survival (OS) between the two groups, including high risk groups, and autoHCT at first response is not recommended.

### Role of transplant in CR2

Two RCTs have evaluated the role of high- dose, myeloablative chemotherapy followed by autoHCT in patients with R/R cHL (18, 19). The British National Lymphoma Foundation (BNLI) conducted a RCT of 40 patients with R/R cHL including 20 primary refractory and 5 multiply resistant patients who were given mini-BEAM as salvage followed by additional mini-BEAM or BEAM followed by autoHCT; enrollment was limited due to patient preference for autoHCT, but the BNLI reported an event free survival (EFS, event being death from any cause or progression) benefit in favor of BEAM followed by autoHCT (3-year EFS 53% vs. 10%, p=0.025) and no statistically significant benefit in OS (p=0.318). Schmitz et al. included 161 R/R cHL patients that received Dexa-BEAM (dexamethasone and carmustine, etoposide, cytarabine, and melphalan), followed by randomization of responding patients to either two additional cycles of Dexa-BEAM or high-dose BEAM and autoHCT. Compared to patients that received Dexa-BEAM only, patients in the autoHCT arm had improved 3-year freedom from treatment failure (34% vs 55%, difference -21%, 95% CI -39.87 to -2.13%, p=0.019). A Cochrane systematic review including meta-analysis of these two trials by Rancea et al. found improvements in progression-free survival (PFS) (HR 0.55, 95% CI 0.35-0.86, p=0.009) and a trend towards an OS benefit for those who underwent autoHCT in CR2 over salvage chemotherapy (7). These findings established autoHCT for responding patients with R/R cHL as the standard of care.

### Factors affecting AutoHCT outcomes

Disease status before transplant is one of the most important factors predicting post- autoHCT outcomes, shown in several retrospective studies. A single-center retrospective study including 153 patients who underwent autoHCT between 1994 and 2003 demonstrated pre-autoHCT CR assessed by functional imaging was associated with twice the rate of EFS compared to those with less than a CR (5-year EFS of 75% and 31% respectively, p<0.0001) (20). An additional single-center retrospective study included 111 patients undergoing autoHCT and showed a pre-transplantation response assessed by PET-CT of CR compared to PR was associated with improved 5-year OS of 90% vs 55% (p=0.001), respectively (21). These differences in outcome persisted through multivariate analysis of pre-transplant disease and patient risk factors (22).

### AutoHCT in older adults

Hodgkin lymphoma has a bimodal age distribution with nearly 20% of patients aged 60 or older at the time of diagnosis. Older patients are more likely to have comorbidities, impaired functional status, and poor social support– factors that may impact transplant eligibility and outcome. The heterogeneity of the older patient population underscores the importance of comprehensive pre-transplant assessment of factors beyond chronological age alone (23).

Validated tools are available to assess a patient’s transplant eligibility beyond chronological age. Comprehensive geriatric assessment across multiple geriatric domains including comorbidities, cognition, and physical functioning performed throughout the transplant period has been shown to optimize transplant tolerability and outcomes (24). The Hematopoietic Cell Transplantation-specific Comorbidity Index (HCT-CI) incorporates variables that impact transplant-related mortality and may be particularly relevant in older patients as it has been shown to be associated with outcomes of autoHCT in retrospective and prospective lymphoma cohorts (25, 26). Lymphoma patients undergoing autoHCT with HCT-CI score of 1-2 or 3 compared to those with HCT-CI of 0 had worse OS, HR 1.23 (95% CI 1.05-1.46, p=0.013) and HR 1.37 (95% CI 1.15-1.16, p<0.0001) respectively.

Previously, a strict upper- age limit was used to exclude older patients from auto-HCT-related clinical trials, and prospective data in this population is limited. In the previously described RCT of autoHCT in R/R cHL by the BNLI, the oldest participant included was 49 years (18), and nearly 10 years later, the other RCT of autoHCT in R/R cHL allowed patients up to age 60 (19). Several retrospective analyses have evaluated post-autoHCT outcomes in patients ≥60 years but are limited by selection bias. Increasingly, transplant centers have proceeded with transplant in older patients. A single-center retrospective study compared outcomes following autoHCT in 15 patients aged 60-67 compared to 137 patients < 60 years old with comparable 2-year OS of 84% and 88% respectively with similar toxicity profiles (27). A multi-center French retrospective registry study of 91 patients aged 60 or older primarily conditioned with BEAM followed by autoHCT demonstrated a 5-year OS of 67% (28). These findings compare favorably to a cohort that included a broad age-range of patients (18–73) and reported a 5-year OS of 79% with BEAM conditioning (29).

Limited data exists for the oldest cHL patients beyond age 70 and primarily consist of small retrospective analyses or underpowered subset analyses of broader cohorts. A retrospective analysis included 346 patients ≥ 60 years with non-Hodgkin lymphoma (NHL) undergoing BEAM conditioning and autoHCT and performed a subset analysis of patients ≥ 70 years (n=67) that showed a nearly 100% incidence of grade 3 toxicities with BEAM conditioning and a 1.71-fold (95% CI, 1.08-2.71) increased risk of progression or death in these patients age ≥ 70 compared to patients under age 70 (30). The authors expressed caution when considering BEAM conditioning in the oldest population undergoing autoHCT.

Despite broadening the definition of transplant-eligibility to include older patients, recent post-autoHCT outcomes have continued to improve (31). With improvements in supportive care and more informed determination of transplant-eligibility through the use of comorbidity scores, autoHCT can be considered in older, frailer, medically complex patients (26, 32).Considering transplant-related risks and treatment toxicities, the availability and efficacy of novel treatments to treat R/R cHL in vulnerable older adults should be considered as well, as the role of BV and/or CPI prior to autoHCT continues to be explored with hopes for durable responses perhaps without the need for transplant.

## Salvage treatments prior to autologous transplant

The primary goal of salvage treatment of patients with R/R cHL prior to autoHCT is to achieve a CR without inducing significant marrow toxicity that would impair hematopoietic stem cell collection. There are multiple conventional salvage regimens used for the treatment of R/R cHL and no one standard exists. Regimens used in the relapsed or refractory setting have traditionally included multi-agent platinum- or gemcitabine-based chemotherapy, and recently, there have been many trials exploring the use of BV and CPI during salvage treatment. These regimens have not been prospectively compared, and choice of regimen is contingent on prior treatment, duration of response to front-line therapy, toxicity profile, and institutional preference.

### Conventional chemotherapy-based salvage regimens

A comprehensive examination of conventional salvage options is beyond the scope of this review but will be briefly discussed. Outcomes of individual salvage regimens are summarized in **Table 1**. Retrospective and phase 2 single-arm studies have described outcomes of conventional salvage regimens, as reviewed by Castagna et al. with overall response rates (ORR) of 68-89% for various platinum-based salvage regimens and hematopoietic stem cell mobilization rates approaching 100% (33). Commonly used platinum-based regimens include DHAP (dexamethasone, cytarabine, cisplatin), ESHAP (etoposide, methylprednisolone, high-dose cytarabine, cisplatin), ICE (ifosfamide, carboplatin, and etoposide), and GDP (gemcitabine, dexamethasone, cisplatin) (34–37). An example of a gemcitabine-based salvage, IGEV (ifosfamide, gemcitabine, vinorelbine), reported a CR rate of 53% with 77% of patients proceeding to transplant (38). Conventional chemotherapy-based salvage regimens are generally effective and well tolerated with primarily hematologic toxicities that can be managed supportively.

**Table 1:** Salvage options for relapsed/refractory classical Hodgkin lymphoma.

Regimen	*n*	ORR (%)	CR (%)	AutoHCT After Salvage (%)	Reference
*Conventional Chemotherapy*					
DHAP	102	89%	21%	N/A	(34)
IGEV	91	81%	54%	70%	(38)
ESHAP	82	67%	50%	N/A	(35)
ICE	65	88%	26%	86%	(36)
GDP	24	70%	17%	100%	(37)
Brentuximab Salvage					
BV Monotherapy	57	75%	43%	56%	(39)
BV-Bendamustine	53	93%	74%	76%	(40)
BV-ICE	45	91%	74%	86%	(42)
BV-IGEV	28	96%	71%	100%	(43)
BV-ESHAP	66	91%	70%	91%	(44)
*CPI Salvage*					
Nivo ± ICE	43	91%	88%	77%	(47)
Pembro-ICE	37	97%	87%	95%	(50)
Pembro-GVD	39	100%	92%	95%	(49)
BV-Nivo	91	85%	67%	74%	(51)

n, number; ORR, overall response rate; CR, complete response; DHAP, dexamethasone, cytarabine, cisplatin; IGEV, ifosfamide, gemcitabine, etoposide, vinorelbine; ESHAP, etoposide, methylprednisolone, cytarabine, cisplatin; ICE, ifosfamide, carboplatin, etoposide; GDP, Gemcitabine, Dexamethasone, and Cisplatin; BV, brentuximab vedotin; Nivo, nivolumab; Pembro, pembrolizumab; GVD, gemcitabine, vinorelbine, liposomal doxorubicin.

### BV-based salvage regimens

Recent retrospective and prospective phase 2 studies incorporating novel agents including BV and CPI are challenging the paradigm of combination chemotherapy as first salvage. BV has been explored as salvage treatment prior to autoHCT as monotherapy (39) or in combination with chemotherapy agents including bendamustine (40, 41), ICE (42), IGEV (43), and ESHAP (44) with ORR of 71-100%, including patients who were previously chemorefractory as summarized in **Table 1**. There is not a clear negative impact on stem cell collection, overlapping toxicities other than myelosuppression and added toxicity of peripheral neuropathy, nor any deleterious impacts on the autoHCT course when BV is used in first-line or beyond treatment regimens. A multicenter retrospective study suggests incorporation of BV into first salvage treatment of patients with high-risk disease features including primary refractory or early relapsing (< 6 months) disease following frontline therapy, may be particularly beneficial (45).

### CPI as salvage

CPIs used alone, sequentially, or in combination with chemotherapy have been explored as first salvage in transplant-eligible patients. The use of conventional chemosensitivity as a predictor of successful autoHCT outcomes may be too narrow and continues to evolve in the CPI era, and a selection of reported salvage regimens including CPI is included in **Table 1**. An intriguing retrospective multi-center study by Merrymen et al. suggests incorporation of a CPI (alone or in combination) in the third line or later salvage setting in transplant-eligible patients may sensitize chemorefractory patients to the high-dose conditioning regimens employed with autoHCT (46). With a median follow up of 19.6 months, 18-month PFS and OS were 81% (95% CI, 69-89) and 96% (95% CI, 87-99) respectively.

A recent single-arm phase 2 prospective study by Mei et al. of nivolumab ± ICE (NICE) evaluated 43 patients with R/R cHL initially with nivolumab monotherapy for 3 biweekly cycles (47). Patients with response (CR or PR in 37 out of 42 patients, 88%) received 3 more cycles of nivolumab followed by autoHCT, while patients with SD or PD received 2 cycles of nivolumab with ICE chemotherapy followed by autoHCT for responding disease. For those with relapsed HL, this treatment paradigm achieved a 93% ORR, and the majority of patients proceeded to transplant.

Retrospective data suggests CPI-exposure may be associated with increased risk of engraftment syndrome, manifesting as high-grade non-infectious fever (≥ 38.5 C) accompanied by rash, diarrhea, and/or transaminitis occurring around the time of neutrophil engraftment in patients undergoing autoHCT. In a retrospective analysis, CPI exposure has been shown to be significantly associated with risk for engraftment syndrome (HR = 8.9, p < 0.001) (48). In the previously mentioned study by Mei et al, 4 of the 33 patients (12%) that proceed to auto-HCT, met pre-specified criteria for engraftment syndrome a median of 8 days after stem cell infusion (47).

In a phase 2 trial of pembrolizumab combined with GVD (Gemcitabine, Vinorelbine, and Liposomal Doxorubicin) followed by autoHCT for responding disease, 68% of patients developed engraftment syndrome at a median of 10 days following stem cell infusion (49). In both prospective studies, engraftment syndrome was successfully managed with early identification, steroids, and supportive care as at worst, engraftment syndrome can manifest as fatal respiratory failure (50). As the use of pre-autoHCT CPI is likely to increase, providers should carefully monitor patients for early signs of engraftment syndrome with prompt initiation of steroids when indicated to mitigate the risk of further complications.

### BV and CPI combinations

Recent 3-year follow up of a single arm phase 1/2 study evaluating a chemotherapy-free regimen of BV with nivolumab as first salvage for transplant-eligible patients with R/R cHL showed favorable post-autoHCT outcomes including a CR rate of 61%, 74% of patients proceeding to autoHCT, and a 91% 3-year PFS (51). Notably, these favorable outcomes were largely driven by patients relapsing 6 months or later from completion of frontline therapy. Consequently, BV with nivolumab has been added to National Comprehensive Cancer Network (NCCN) guidelines as a potential first salvage regimen for transplant-eligible patients.

In the initial phase 2 FDA registration trial of BV monotherapy for patients with cHL relapsed after autoHCT (8), 9% of patients (9 of 102) remained in CR at 5 years after 16 cycles of BV without any additional therapy (52), suggesting a subset of patients may not benefit from high-dose consolidative approaches. An ongoing clinical trial of BV and CPI in patients with R/R cHL and deferral of autoHCT is being explored (NCT04561206). Further work is needed to define the subset of patients who achieve durable response to BV without the need for consolidative autoHCT.

### Peri-transplant radiation therapy

The role of peri-transplant radiation therapy (RT) has not been explored in RCT, and its use is largely supported by single-arm prospective and retrospective studies and expert panel recommendations. The International Lymphoma Radiation Oncology Group published guidelines regarding the use of RT for cHL advising that RT may play a role in transplant-eligible patients with limited sites of refractory disease, bulky disease, or as post-transplant consolidation for those with a PR at the time of autoHCT (53).

A single-center retrospective study evaluated 80 consecutive patients with R/R cHL who received post-transplant consolidative involved field radiation therapy (IFRT) for lymph nodes ≥ 2 cm (needed in 40% of 80 patients) with a 2-year PFS benefit noted for those who underwent RT (67% vs. 42%, p < 0.01) but no 2-year OS benefit (54). An additional single-center retrospective study evaluated outcomes of R/R cHL patients who received IFRT in the immediate post-transplant period for residual disease (identified on post autoHCT PET/CT) and showed a 5-year OS of approximately 80%; however, when radiation was used for late progression of disease after autoHCT (beyond transplant day +100) limited success was achieved with a 5-year OS of 30% (55).

There is also some excitement about the synergy of radiation and immunotherapy as a means to increase rates of CR prior to autoHCT. Radiation is being explored in R/R cHL patients with disease progression on CPI in an ongoing clinical trial (NCT03480334). Early results have shown half of patients with tumor response even outside the radiation field while continued on CPI showing how RT can re-sensitive patients to immunotherapy (56). In summary, RT remains reasonable to consider for limited sites of disease and for any sites of peri-transplant residual PET-positive disease.

## Conditioning regimens for autologous transplants

Common conditioning regimens used for autoHCT in cHL patients include BEAM (carmustine, etoposide, cytarabine, and melphalan), CBV (cyclophosphamide, carmustine, and etoposide), and GemBuMel (gemcitabine, busulfan, and melphalan) (57, 58), and no standard exists. Toxicities vary between regimens and can help clinicians choose appropriate regimens; notably, high dose carmustine and busulfan can both put patients at risk for pulmonary toxicity and nephrotoxicity, whereas melphalan’s dose limiting toxicities are diarrhea and mucositis.

A CIBMTR analysis including 1,012 cHL patients undergoing autoHCT from 1995-2008 evaluated the impact of common conditioning regimens on outcome (59). After stratifying for conditioning regimen, the probability of 3-year OS for BEAM, CBV, BuCy and TBI were 79%, 68-73%, 65% and 47%, respectively (p<0.001 between all groups), and BEAM was associated with improved OS in multivariate analysis. Compared with BEAM, CBV, BuCy and TBI were associated with increased mortality (CBV vs. BEAM, HR 1.53, p=0.003; BuCy vs. BEAM, HR 1.77, p<0.001; TBI vs. BEAM, HR 3.38, p<0.001). One -year TRM was 4-8% in the entire cohort, with no statistically significant differences between conditioning regimens.

Considering the pulmonary toxicity, cost, and occasional shortages of carmustine, alternative regimens without carmustine include BeEAM (bendamustine, etoposide, cytarabine, and melphalan) and thiotepa-based TEAM (thiotepa, etoposide, cytarabine, melphalan) and TECAM (thiotepa, etoposide, cytarabine, cyclophosphamide, and melphalan) have been evaluated. BeEAM was studied in a phase 1/2 trial including 15 cHL patients undergoing autoHCT and demonstrated engraftment at a median of 10 days, neutropenic fever in approximately half of the patients, and median disease-free survival of 19 months for the R/R cHL cohort (60). A multi-center retrospective study compared TEAM and BEAM, finding similar post-transplant outcomes in the two groups (PFS HR 1.15 (95% CI 0.7-1.87, p=0.59) (61). A prospective study of TEAM demonstrated a 1-year non-relapse mortality of 3.3% and median time to engraftment of 12 days (62). TECAM was studied by single-center retrospective study of 120 patients with the same time to engraftment compared to BEAM (median 11 days for each cohort) and 3-year PFS for TECAM vs. BEAM of 49% vs. 62% (p=0.16) (63). In summary, the various conditioning regimens for autoHCT have not been compared in randomized trials, and thus, selection is largely based on patient factors, institutional preference, and toxicity profile.

### Outpatient AutoHCT

AutoHCT with BEAM conditioning is traditionally administered in the inpatient setting, but single-center retrospective studies suggest outpatient administration of BEAM and hematopoietic stem cells with close monitoring is safe, feasible and does not compromise outcomes (64, 65). Patients may be admitted later in the transplant course for cytopenias needing frequent transfusions or infectious complications and neutropenic fever. Overall, there is reduction in length of inpatient stay, reduced costs, and lower rates of infections and enteritis with outpatient autoHCT (64). Some patients who experience minimal complications can remain completely outpatient throughout the transplant course with a median of 13 hospital days avoided per patient for outpatient BEAM compared to historical controls (66).

In a meta-analysis of the outcomes of 740 outpatient autoHCT across 9 studies for any disease indication, those who underwent outpatient autoHCT had significantly lower risk of neutropenic fever (pooled OR 0.55, 95% CI 0.29-0.65, p<0.001) with no statistically significant difference in OS in the 3 studies that reported survival outcomes (67). Outpatient autoHCT programs take advantage of improvements in the supportive care including infectious prophylaxis, growth factors, robust transfusion support, and oral care regimens. Many of these outpatient transplant programs needed to be placed on hold or substantially modified with the ongoing SARS-CoV-2 pandemic.

### Alternative and adjunctive conditioning strategies

Other agents have been explored as adjunctive to conditioning chemotherapy. Rituximab with BEAM has been reported in a single-center retrospective study with 78% disease-free survival at 3 years, 94% 3-year OS, and no impact on outcome by CD20 status suggesting perhaps multiple mechanisms of action (68). Tandem autoHCT comprising of conditioning with two different myeloablative regimens, such as high-dose melphalan for first autoHCT followed by BEAM or TBI conditioning for second autoHCT, has been explored in high-risk patients in risk-adapted, non-randomized trials to improve long-term autoHCT outcomes (69–71). Interpretation of outcomes is limited at present with maintenance treatment after autoHCT now commonplace for these high-risk patients. Tandem autologous transplant has fallen out of favor and is not recommended by the American Society for Blood and Marrow Transplantation (72).

While TBI conditioning was used in 545 patients included in the previously mentioned CIBMTR analysis (59), radiation plays a limited role in conditioning regimens employed in autoHCT for cHL patients with concerns for cardiac and pulmonary toxicity and risks of secondary malignancies. TBI as a part of conditioning has been shown to have increased risk of overall 2-year mortality (HR 1.51, 95% CI 1.09-2.08, p=0.013) and 2-year NRM (HR 1.51, 95% CI 1.01-2.25, p=0.43) based on an additional analysis of CIBMTR data (73). TBI should only be considered in select circumstances in which patients remain in PR involving extranodal and/or bone marrow sites despite all available salvage options (53).

Additions to standard conditioning regimens are undergoing evaluation to deepen consolidation responses and reduce the risk of relapse after autoHCT with a focus on cellular and immune-based therapies. Radioimmunotherapies, using an antibody labeled radionuclide to target the radiation’s effects, like ^131^Iodine-tositumumab and ^90^Yttrium-ibritumomab tiuxetan have been studied but are not commonly used due to the complexity of administration, delayed engraftment, and prolonged cytopenias (74). An ongoing phase 2 trial evaluating a novel anti-CD25 radioimmunotherapy (^90^Yttrium-basiliximab) combined with BEAM conditioning (aTAC-BEAM) is ongoing (NCT04871607). One approach was shared in a recent abstract with the outcomes of a single-center single-arm pilot study of tandem autoHCT and anti-CD30 chimeric antigen receptor (CAR) T-cell infusion in several especially high-risk and chemorefractory patients highlighting the potential future role of cellular therapy in this space (75). Another upcoming trial will add isatuximab, a CD38 antibody, during collection and transplant to modulate the immune system, improving T-cell recovery, to reduce changes of relapse after autoHCT (NCT05346809). Improvements in supportive care are also being explored, like the addition of romiplostim to reduce need for platelet transfusions (NCT04478123) and allogeneic engineered human endothelial cells to address diffuse injury to vascular stem cells during autoHCT (NCT05181540).

## Maintenance therapies after autologous transplant

Despite the success of consolidative autoHCT in approximately half of patients with R/R cHL, a substantial subset of patients will ultimately relapse (19, 76). Retrospective studies have sought to identify patient- and disease-related characteristics predictive of post-autoHCT relapse. In a recent retrospective study of 501 patients who underwent autoHCT at MD Anderson Cancer Center from 2005 to 2019, Nieto et al. found the following factors independently associated with poorer PFS after autoHCT in multivariate analysis: primary refractory disease (HR 1.41, 95% CI 1.01-1.97, p=0.04), more than two prior lines of therapy (HR 1.60, 95% CI 1.08-2.36, p=0.01), bulky relapse (HR 1.56, 95% CI 1.15-2.12, p=0.004), B-symptoms at relapse (HR 1.68, 95% CI 1.19-2.37, p=0.003), and a positive PET at autoHCT (HR 2.60, 95% CI 1.83-3.69, p<0.0001) (77). Other groups are exploring integration of pre-transplant Deauville score on PET scan and residual metabolic tumor volume in determining a patient’s risk of relapse post-autoHCT (78). Knowing the risk factors that are associated with poorer outcomes, we can identify patients who may benefit from more aggressive or additional treatment including post-autoHCT maintenance.

### BV maintenance

The concept of maintenance therapy post-autoHCT aims to reduce the risk of relapse without adding substantial toxicity. BV has been considered an acceptable candidate for post-autoHCT maintenance due to its known monotherapy activity in relapsed setting and reasonable toxicity profile (52).

The sentinel trial of post-autoHCT maintenance therapy in cHL was the randomized, double-blind, placebo-controlled phase 3 AETHERA trial in which BV monotherapy was administered for up to 16 cycles post-autoHCT (1.8 mg/kg every 3 weeks starting 30-60 days post-autoHCT) in patients with high-risk features including primary refractory cHL, early relapsed cHL (defined as remission duration of less than 12 months), or extranodal involvement at the start of pre-transplantation salvage chemotherapy (79). Prior BV exposure was not allowed. A total of 329 patients with at least one of these defined high-risk features were randomized to receive up to 16 cycles of BV (n=165) or placebo (n=164). With a median follow-up time of 30 months, PFS was superior in the BV group compared to placebo group (42.9 months vs. 24.1 months, respectively, p=0.0013) (79). There was more peripheral neuropathy (PN) in the BV group (67% vs. 10%), and the most common grade 3 adverse events higher in the BV group compared to placebo included neutropenia (29% vs. 10%), peripheral sensory neuropathy (10% vs. 1%), and peripheral motor neuropathy (6% vs. 1%); thus, dose modifications were needed in 32% of patients receiving BV maintenance and led to discontinuation of treatment in 23% of patients. The median time to onset of PN was 13.7 weeks.

Subsequent longer-term follow up of the AETHERA trial was published that continued to show a PFS benefit for BV maintenance after autoHCT compared to placebo (5-year PFS of 59% for BV vs. 41% placebo) (80). In long-term follow-up, 73% of patients in the BV arm reported complete resolution of PN, with median time to resolution of PN of 37.6 weeks. There has been no OS benefit reported on the trial with significant post-protocol cross over as many patients on the placebo arm received BV after relapse. Similar results were confirmed in real-world studies as well outside of the regulated clinical trial setting calling into question the optimal timing of BV as salvage therapy before or after transplant and its role as post-transplant maintenance (81, 82).

In addition to a lack of clear OS benefit and added incidence of PN with post-autoHCT BV maintenance for many patients who would otherwise be cured of their cHL, one study estimated significant financial toxicity of BV maintenance with an estimate of $148,664/QALY gained as maintenance therapy compared to use of BV salvage treatment at time of relapse (83). This value exceeds the accepted cost-effectiveness threshold outside of the US healthcare system, limiting its widespread adoption. There is also concern raised about the increased immunosuppression and its infection risk putting patients at risk for life-threatening respiratory infections including *Pneumocystis jiroveci* pneumonia. One center reported an incidence of PJP of 4.1% in 14 of 339 patients receiving BV without PJP prophylaxis (not currently recommended), above a commonly accepted threshold of 3.5% incidence for recommending PJP prophylaxis (84).

A single-center retrospective analysis of 20 patients with R/R cHL in a resource-limited where patients bared full responsibility for drug costs reported outcomes of fewer cycles of BV maintenance compared to AETHERA (4 vs. 16, respectively). With a median follow up of 26.5 months, the cohort demonstrated a 2-year PFS of 72%, and median PFS was not reached (85). While the number of patients in this single study was small, these outcomes compare similarly to those demonstrated in AETHERA and call into question the total number of maintenance BV cycles needed to improve outcomes. Consensus guidelines continue to recommend 16 cycles of BV maintenance in BV-naïve cHL patients with high-risk features following autoHCT (86).

### CPI and other maintenance after AutoHCT

With CPI’s emerging role as a highly effective salvage agent in HL and building on the success of PD-1 blockade as adjuvant treatment in select solid malignancies, CPIs are being investigated as a maintenance strategy after autoHCT in HL. Armand et al. conducted a multicenter phase 2 trial evaluating pembrolizumab administered for 8 cycles after autoHCT in 30 patients with R/R cHL (87). Pre-planned analysis of 18-month PFS and OS were 82% and 100%, respectively. Pembrolizumab maintenance was associated with a 43% prevalence of immune related adverse events (irAE); the most common grade 2 or higher irAE was pneumonitis/cough in 5 patients. Similarly, nivolumab is being evaluated as maintenance therapy post-autoHCT for 6 months in a phase 2 trial that has reported preliminary results in 37 patients (out of a planned enrollment of 40 patients). With a median follow up of 9.2 months, 1-year OS was 100%, and 46% of patients experienced an treatment-related AE (88).

The combination of BV with nivolumab has been explored as another consolidation strategy for patients who are not previously refractory to both drugs. This phase 2 trial (NCT03057795) completed enrollment of 65 patients in 2021 and so far, has been reported to be an efficacious regimen with only 1 relapse event in 59 patients after 15 months of median follow-up after autoHCT (89). Longer-term follow up will further clarify efficacy and tolerability. There is an ongoing Phase I trial of CD30 CAR-T when given between 14 and 20 days following autoHCT around the time of count recovery (NCT02663297). Lenalidomide maintenance for up to 18 months after autoHCT had also been explored but was limited by grade 3 to 4 hematologic toxicity in more than half of participants (90). Polatuzumab Vedotin is also being explored as maintenance in CD20+ cHL as well as other NHL (NCT04491370).

We continue to hope to find tolerable and effective maintenance options for patients at the highest risk of relapse who will see the most benefit.

## The role of allogenic transplant

Allogeneic hematopoietic stem cell transplantation can provide durable responses in a subset of medically fit patients with R/R cHL who require multiple lines of therapy to achieve response, have a best response of PR to salvage CPI, or relapse after autoHCT. The anti-lymphoma properties of alloHCT are primarily mediated through a graft versus lymphoma (GVL) effect induced by alloreactive donor T-cells (91, 92), and this approach can provide long-term survival in 25-40% of carefully selected patients (93–95).

A retrospective analysis by the EBMT (European Group for Blood & Marrow Transplantation) included 2,204 patients with R/R cHL who underwent alloHCT for both chemosensitive (CR or PR) or chemorefractory (SD or PD) disease and suggests improved outcomes with more contemporary transplant practices (3-year OS: 1990-1994, 21% vs. 2011-2014, 61%) with an increase in the use of haploidentical donors, reduced intensity conditioning (RIC), and peripheral blood as stem cell source over time (96). While alloHCT can be powerful therapeutic tool, its application is limited by the associated toxicities, primarily driven by regimen-related adverse effects, graft vs. host disease (GVHD) and need for long-term immunosuppression with the associated infectious risk. In this EBMT analysis, rates of 1-year non-relapse mortality (NRM) have fallen from 58% (95% CI 39-73%) in the 1990s to 19% as of 2011-2014 (95% CI 16-21%) and rates of severe grade 3-4 GVHD at 50 days has fallen from 25% (95% CI 11-42%) in the 1990s to 8% as of 2011-2014 (95% CI 6-10%).

### Disease status prior to AlloHCT

Depth of response impacts alloHCT outcomes with more favorable outcomes observed in those achieving a pre-transplantation CR. A CIBMTR analysis that included 1,694 patients with R/R HL undergoing alloHCT between 2009 and 2019 showed improved rates of 3-year OS in patients with a pre-transplantation CR or PR compared to those deemed chemoresistant (SD or PD) (67% vs. 50%, p<0.0001) (12). Similarly, a multi-center retrospective analysis from GATMO (Grupo Argentino de Transplante de Médula Ósea) that included 113 patients with R/R cHL undergoing alloHCT showed that CR as compared to PR or SD/PD at the time of transplantation was associated with improved OS in univariate analysis (2-year OS for CR 57%, PR 41%, SD/PD 13%, p=0.001) and was the only variable predictive of OS in multivariate analysis (p=0.002) (97).

For patients who are refractory to chemotherapy and with progression after BV and CPI containing regimens, there is no standard of care, and enrollment of patients on clinical trials is advised to achieve disease response prior to alloHCT. Given the progress of chimeric antigen receptor T-cells (CAR-T) in NHL, there are several ongoing trials of CD30 directed CAR-T in cHL (RELY-30, CHARIOT) (98, 99) and preclinical investigations of bispecific antibodies. AlloHCT remains a potentially curative options for even the most difficult to treat patients.

### Donor selection for AlloHCT for cHL

The traditional approach for donor selection has emphasized use of fully matched-related or-unrelated donors due to the associated low rates of graft failure and more favorable rates of GVHD compared to mismatched donors (100–102). However, only about 30% of patients have a fully matched-related donor (MRD), and rates of identifying a fully matched-unrelated donor (MUD) vary considerable by racial and ethnic group in the U.S. population from Black South or Central American at 16% to White Europeans at 75% (103). The introduction of umbilical cord blood (UCB) hematopoietic stem cell donors in the early 1990s led to alloHCT in an increasing number of lymphoma patients, but prolonged time to engraftment (time to neutrophils engraftment at 28 days, MUD 94% vs. UCB 66%, p<0.001) and associated increased infection risk have limited this approach (104). The use of post-transplant cyclophosphamide (PTCy) has allowed for use of haploidentical donors, and a substantial increase in the donor pool. The impacts of donor type on cHL alloHCT outcomes continues to evolve (**Table 2**).

**Table 2:** Donor selection for allogenic hematopoietic cell transplant in R/R cHL.

Time	Donor Sources	OS	Grade 2-4 Acute GVHD (Day+100), Cumulative Incidence (%)	Chronic GVHD, Cumulative Incidence (%)	Reference
1998-2007	MRD, n=38 MUD, n=24 Haplo, n=28	*2-Year OS:* MRD: 53% MUD: 58% Haplo: 58%	MRD: 50% MUD: 50% Haplo: 43%	*2-year CI:* MRD: 50% MUD: 63% Haplo: 35%	(105)
2000-2017	MRD, n=68 MUD, n=21 Haplo, n=24	*2-year OS:* MRD: 44% MUD: 15% Haplo: 70%	Entire Cohort: 15%	*1-year CI:* Entire Cohort: 24%	(97)
2008-2013*	MRD, n=807 Haplo, n=180	*3-year OS:* MRD: 62% Haplo: 61%	MRD: 25% Haplo: 27%	*1-year CI:* MRD: 45% Haplo: 12%	(106)
2009-2012	MRD/MUD, n=133 Haplo, n=65	*2-year OS:* MRD/MUD: 63% Haplo: 67%	MRD/MUD: 16% Haplo: 15%	*1-year CI:* MRD/MUD: 32% Haplo: 18%	(107)
2009-2016*	Haplo BM, n=357 Haplo PB, n=169 UCB, n=214	*4-Year OS:* Haplo BM: 58% Haplo PB: 59% UCB: 49%	Haplo BM: 20% Haplo PB: 35% UCB: 43%	*4-year CI:* Haplo BM: 24% Haplo PB: 32% UCB: 28%	(108)
2010-2013	MRD, n=338 MUD, n=273 Haplo, n=98	*2-Year OS:* MRD: 71% MUD: 62% Haplo: 67%	MRD: 18% MUD: 30% Haplo: 33%	*1-year CI:* MRD: 25% MUD: 41% Haplo: 26%	(109)

GVHD, Graft-Versus-Host Disease; OS, Overall Survival; MRD, matched related donor; MUD, matched unrelated donor; Haplo, haploidentical donor; BM, bone marrow; PB, peripheral blood; UCB, umbilical cord blood.

*Analysis also included patients with NHL.

Prospective data to guide donor selection when multiple donors are available are lacking. Mounting retrospective data supports prioritization of a fully matched donor when available followed by a haploidentical donor.

A CIBMTR analysis of 987 patients with both R/R cHL (n=222, 22.5%) and NHL (n=765, 77.5%) undergoing alloHCT between 2008 and 2013 showed no significant differences in outcome for MRD compared to haploidentical donors for NRM (p=0.06), PFS (p=0.83), OS (p=0.34) and cumulative incidence of day 100 grade 2-4 acute GVHD (27% vs. 25%; p=0.84) (106). However, chronic GVHD at 1 year was significantly lower after haploidentical compared to MRD transplantation (12% vs. 45%; p<0.001). Analysis of an EBMT cHL cohort undergoing haploidentical or MUD transplantation between 2010 and 2013 described similar findings of equivalent OS and lower risk of 1-year chronic GVHD for haploidentical donors (26% vs. 40%, respectively; p=0.04) (109).

With the use of haploidentical donors and PTCy, UCB donors have played a diminishing role as a source for hematopoietic stem cells. A retrospective analysis using pooled data from the CIBMTR, LWP-EBMT, and Eurocord evaluated 457 NHL and 283 HL patients undergoing haploidentical and UCB alloHCT and showed improved outcomes with haploidentical compared to UCB donors (4- year OS, 58-59% vs. 49%, respectively, p=0.008) and grade 2-4 acute GVHD (43% vs. 20% respectively, p<0.0001) (108). An RCT comparing haplo identical vs. double UCB transplantation following RIC in patients with hematologic malignancies included limited numbers of patients with HL (18 out of 368 total patients) and showed improved 2-year OS with haploidentical donors (haplo 57% vs. dUCB 46%, p=0.04) (110). These findings suggest haploidentical transplantation with PTCy as a more favorable alternative donor approach.

### Conditioning for AlloHCT in cHL

There is no standard conditioning regimen for alloHCT in HL, and both myeloablative conditioning (MAC) and nonmyeloablative/reduced intensity conditioning (RIC) are used. Older data describing transplants prior to 1996 suggested intensifying conditioning did not improve outcomes (94). In the early 2000s, there was considerable mortality risks for either–MAC with 1-year TRM of 22% (111) and RIC with 1-year TRM of 30% (112). The EBMT performed a retrospective analysis including 168 cHL patients undergoing alloHCT between 1997 and 2001 analyzed by conditioning strategy (RIC, n = 89; MAC, n = 79) that showed an OS benefit in patients receiving RIC (5-year OS 28% vs. 22%, HR 1.62, p=0.04) (113).

A more contemporary retrospective analysis by the EBMT has challenged their earlier findings. Genadieva-Stavrik et al. retrospectively evaluated the impact of conditioning intensity in 312 HL patients undergoing alloHCT between 2006 and 2010 (RIC, n=249; MAC, n=63). With a median follow-up of 56 months, there were no significant 2-year OS differences between RIC and MAC (62% vs. 73%, respectively, p=0.13) (114). The disparate findings of the 2 EBMT cohorts are likely due to improvements in transplant strategies, patient selection, and supportive care. Additionally, the choice of a specific RIC regimen did not have a clear impact on risk of relapse or OS based on a recent CIBMTR analysis of 3 common RIC regimens used in HL (fludarabine/busulfan, fludarabine/melphalan, or fludarabine/cyclophosphamide) (115). These findings suggest the selection of conditioning regimens must take multiple patient-, disease- and donor-related factors into account, with no single best approach for all patients.

### Use of CPI before AlloHCT

The use of CPI in R/R cHL prior to alloHCT poses unique challenges. Fatal hyper-acute GVHD (diagnosed within 14 days after transplantation), non-infectious febrile episodes and other immune-related adverse events have been noted in the peri-alloHCT period in CPI-exposed patients, owing to the long CPI half-life and associated activation of cytokines and CD4+ helper T lymphocytes (116–118). A 2019 meta-analysis by Ijaz et al. evaluated 283 subjects that received peri-transplant CPIs and showed that of the 107 subjects with pre-alloHCT CPI exposure, 7% developed hyperacute GVHD, 56% developed acute grade 2-4 GVHD, and 29% developed chronic GVHD (117). A 2018 pooled analysis by Dada and Usman showed higher rates of grade 3-4 acute GVHD in patients with pre-alloHCT CPI exposure compared to historical controls with no CPI exposure (28% vs. 8%, p=0.02) (119).

Efforts are ongoing to identify risk-mitigation strategies for patients undergoing alloHCT after PD1-blockade. In an international multi-center retrospective study, longer time interval (> 80 days) between CPI and alloHCT was associated with less frequent severe (grade 3-4) GVHD (HR 0.4, p=0.01) (120). GVHD prophylaxis with PTCy was associated with improved 2 year-PFS compared to no PTCy (80% for haplo donor with PTCy, 74% for non-haplo donor with PTCy vs. 60% for non-haplo donor without PTCy respectively, p=0.028). Nivolumab was found in blood samples of alloHCT patients up to 56 days after its last dose, and the T-cell activation due to ongoing nivolumab effects are able to be mitigated by PTCy (121). In a Japanese cohort, PTCy reduced GVHD incidence in CPI-exposed patients from 58% to 15% (122). Considering the aforementioned, the use of PTCy as GVHD prophylaxis is recommended for all patients with CPI exposure prior to alloHCT to mitigate increased risk of GVHD and improve outcomes.

### AlloHCT as first transplant

Limited data exists for patients who proceed with alloHCT in first response. This approach has been considered for patients with PR as best response or those with highly refractory disease requiring multiple lines of therapy. The LWP-EBMT evaluated outcomes of 190 patients from 2000 to 2016 with high-risk disease defined as receipt of three of more lines of treatment (59% with CR or PR prior to alloHCT, 22% with prior CPI exposure), undergoing a 10/10 MRD or MUD alloHCT as first transplant (123). One-year NRM and 3-year OS were 19% (95% CI 14–26%) and 58% (95% CI 51-66%), respectively. Multivariable regression analysis identified MAC with T-cell depletion as an independent predictor of improved OS; recipient age and female donor to male recipient were found to be predictors of worse OS. For a select subset of patients having exhausted available therapies and lacking clinical trial options, alloHCT with MAC is a potentially curative treatment option.

We continue to explore new strategies to improve alloHCT outcomes; building on the experience of maintenance BV in the autoHCT setting, post-alloHCT BV maintenance is undergoing evaluation as well (NCT03540849, NCT03652441).

## Conclusions

Despite significant advancements in the management of HL, hematopoietic stem cell transplantation continues to play an important therapeutic role for patients with relapsed/refractory disease. Enhanced supportive care, less toxic conditioning regimens and post-transplant maintenance have improved transplant outcomes. With the addition of novel, efficacious therapies in earlier lines of cHL treatment, the role of hematopoietic stem cell transplantation is evolving. Mounting data suggests a chemo-sensitizing effect of pre-transplant PD-1 blockade that may allow for broader use of autologous SCT and attenuation of alloHCT in heavily pre-treated patients. For the subset of patients considered for alloHCT, haploidentical donors have expanded the donor pool and post-transplant cyclophosphamide-based GVHD prophylactic regimens have improved outcomes for haploidentical transplantation and CPI-exposed patients. With the incorporation of combination chemotherapy, novel agents and hematopoietic stem cell transplantation, the majority of patients with R/R cHL enjoy favorable outcomes.

## Author contributions

All authors listed have made a substantial, direct, and intellectual contribution to the work and approved it for publication.
